# Immunohistochemical expression of mitochondrial membrane complexes (MMCs) I, III, IV and V in malignant and benign periampullary epithelium: a potential target for drug therapy of periampullary cancer?

**DOI:** 10.1186/1471-2407-10-80

**Published:** 2010-03-04

**Authors:** Mark M Aloysius, Abed M Zaitoun, Timothy E Bates, Mohammad Ilyas, Dumitru Constantin-Teodosiu, Brian J Rowlands, Dileep N Lobo

**Affiliations:** 1 Division of Gastrointestinal Surgery, Nottingham Digestive Diseases Centre, NIHR Biomedical Research Unit, Nottingham University Hospitals, Queen's Medical Centre, Nottingham NG7 2UH, UK; 2 Department of Pathology, Nottingham Digestive Diseases Centre, NIHR Biomedical Research Unit, Nottingham University Hospitals, Queen's Medical Centre, Nottingham NG7 2UH, UK; 3 New-Use Therapeutics Limited, BioCity Nottingham, Pennyfoot Street, Nottingham NG1 1GF, UK; 4 School of Biomedical Sciences, University of Nottingham, Nottingham NG7 2UH, UK

## Abstract

**Background:**

Mitochondrial membrane complexes (MMCs) are key mediators of cellular oxidative phosphorylation, and inhibiting them could lead to cell death. No published data are available on the relative abundance of MMCs in different periampullary cancers. Therefore, we studied the expression profile of MMCs I, III, IV and V in periampullary cancers, reactive pancreatitis, normal pancreas and chronic pancreatitis.

**Methods:**

This was a retrospective study on tissue microarrays constructed from formalin-fixed paraffin-embedded tissue from 126 consecutive patients (cancer = 104, chronic pancreatitis = 22) undergoing pancreatic resections between June 2001 and June 2006. 78 specimens of chronic pancreatitis tissue were obtained adjacent to areas of cancer. Normal pancreatic tissue was obtained from the resection specimens in a total of 30 patients. Metastatic tumours in 61 regional lymph nodes from 61 patients were also studied.

**Results:**

MMCs I, III, IV and V were highly expressed (p < 0.05) in all primary periampullary cancers compared with metastatic lymph nodes and adjacent benign pancreas. MMCs III, IV and V were highly expressed in all cancers regardless of type compared with chronic pancreatitis (p < 0.05). Higher expression of MMCs I and V was associated with better survival and may, in part, relate to lower expression of these MMCs in poorly differentiated tumours compared with well and moderately differentiated tumours.

**Conclusions:**

Differential expression of MMCs III, IV and V in primary periampullary cancers compared with adjacent benign periampullary tissue and chronic pancreatitis is a novel finding, which may render them attractive anticancer targets.

## Background

The mitochondrial respiratory chain complexes or mitochondrial membrane complexes (MMCs) I, II, III and IV are proteins responsible for electron transport and the associated proton pumping which generates a proton gradient and mitochondrial membrane potential which is then used (via ATP synthase, complex V) to generate adenosine-5'-triphosphate (ATP), the central energy currency of the cell. Mitochondria also play a key role in apoptosis, through the modulation of membrane potential and the co-ordinated release of mitochondrial proteins such as cytochrome *c *[1]. Mitochondrial complexes have also been shown to be specific targets for drug therapy in cancer and drugs called "mitocans" [2-5] can disrupt the integrity of mitochondria causing cytosolic release of modulators of apoptosis and activation of mitochondria-dependent cell death signalling pathways inside cancer cells [6-8].

Although the concept of mitocans as novel anticancer agents is interesting, their safety and efficacy are dependent on their selectivity in targeting malignant tissue over normal tissue in inducing apoptosis [7,9-11]. A key approach to targeting mitochondrial components involves blocking the electron transport chain by inhibiting MMCs I, II, III, IV and V [11,12]. What is key to effecting some paradigms of mitochondrially-mediated apoptosis is the ability to induce a sufficiently large decrease in mitochondrial membrane potential, which may be made possible by inhibiting any one specific mitochondrial membrane.

Several classes of drugs are capable of achieving this *in vitro *[12-15], but before these agents are investigated clinically in specific cancers it is critical to ascertain the relative expression of these target membrane complexes in malignant tissue compared to benign tissue. Such a mechanistic approach would enable selection of mitocans that are target specific with least toxicity and optimal efficacy.

Periampullary cancer is a collective term used for cancers arising from or near the ampulla of Vater and include ampullary cancers, duodenal cancers, pancreatic head cancer and distal cholangiocarcinoma. As the relative expression of MMCs in malignant and benign pancreatic epithelium has not yet been characterized, we performed this study to determine the immunohistochemical expression of MMCs in tissue microarrays (TMAs) of formalin-fixed archived specimens of periampullary cancers (pancreatic ductal adenocarcinoma, ampullary adenocarcinoma, cholangiocarcinoma and duodenal adenocarcinoma), chronic pancreatitis, and normal pancreatic tissue, and to determine any association of MMC expression in cancer with survival.

## Methods

### Study design, setting and ethics

This immunohistochemical study was performed on archived formalin-fixed pancreatic tissues from all patients who underwent pancreatic resections between June 2001 and June 2006 at Nottingham University Hospitals, Queen's Medical Centre, Nottingham, UK. The Ethics Committee of Nottingham University Hospitals approved the conduct of this study.

### Subjects

We included 126 consecutive patients who underwent pancreatic resections (104 for cancer and 22 for chronic pancreatitis) [16]. Additional specimens of chronic pancreatitis tissue were obtained from areas of inflammation adjacent to cancers. Normal pancreatic tissue was obtained from the resection specimens, adjacent to areas of cancer or chronic pancreatitis. Metastatic lymph nodes from patients with cancers were also studied.

### Construction of tissue microarrays

Formalin-fixed, paraffin-embedded tissue blocks containing pancreatic ductal adenocarcinoma, ampullary adenocarcinoma, cholangiocarcinoma, duodenal adenocarcinoma, chronic pancreatitis and normal pancreatic tissue were identified on haematoxylin and eosin stained slides, marked by a single histopathologist (AMZ) and tissue microarrays (TMAs) were constructed in triplicate by a method previously used by us [16].

### Immunohistochemical staining

Slides were deparaffinized in xylene, then hydrated via graded dilutions of alcohol followed by running tap water. Following a rinse in de-ionized water, the sections were pre-treated with boiling sodium citrate 10 mM pH 6.0 for 23 min to unmask antigenicity. Subsequently, a Labelled Streptavidin Biotin (LSAB) immunoperoxidase procedure was performed using 3,3'-diaminobenzidine (DAB) as the chromogen on a TechMate 500+ automated stainer (Dako UK Ltd., Ely, UK). The staining protocol consisted of incubating the sections with 0.3% hydrogen peroxide for 10 min to block endogenous peroxidase, washing in phosphate buffered saline (PBS) buffer, and incubation in goat serum for 20 min. After washing, the slides were incubated with mouse anti-mitochondrial, OXPHOS complexes I, III, IV and V antibodies (Invitrogen, Paisley, UK) at an optimal dilution of 1:20, 1:20, 1:20 and 1:100, respectively, in an antibody diluent (S2022; Dako) for 1 h at room temperature, followed by further washing in PBS. Sections were then incubated with a biotin linked secondary antibody for 30 min followed by washing in PBS and then streptavidin-horseradish peroxidase (30 min) using a reagent kit from Dako (K5001). Immunostaining was visualized using DAB present in the above kit for 10 min, followed by light counterstaining with haematoxylin. Negative controls were also performed on TMAs using diluent only. The control TMAs did not stain positively, confirming the positive staining observed using this protocol was that of MMCs I, III, IV and V. Liver sections were used as positive controls.

### Immunohistochemical scoring

MMC expression was calculated by combining an estimate of the percentage of immunoreactive cells (quantity score) with an estimate of the staining intensity (staining intensity score) as follows: no staining was scored as 0, 1-10% of cells with positive staining were scored as 1, 10-50% as 2, 50-70% as 3, and 70-100% as 4. Staining intensity was rated on a scale of 0 to 3 as follows: 0 = negative (no colour); 1 = weak brown, 2 = moderate brown, and 3 = strong brown. The raw data were converted to the immunohistochemical score (IHS) by multiplying the quantity and staining intensity scores. Therefore, the score could range from 0 to 12. An IHS ≥ 2 was considered as positive expression [17]. Staining was further categorized as high (score >8) or low (score ≤ 8), based on the median staining score of 8 for all MMCs. Immunohistochemical scoring was performed independently by two observers (MMA and AMZ) and the inter-observer variability was <3%.

### Proliferative capacity

Proliferative capacity of the cancers was evaluated using a well established method and represented as the volume corrected mitotic index (VCMI) [18]. This was also performed by 2 independent observers (MMA and AMZ) and the inter-observer variability was <3%.

### Statistics

Statistical analysis was performed using SPSS v16.0 for Mac (SPSS Inc., Chicago, IL, USA). Means were compared using the Student t-test. One way ANOVA was used when comparing 3 or more groups. Results were considered significant at p < 0.05. Correlations were performed using Pearson's correlation test. Survival analysis was carried out using the Kaplan-Meier method with log rank Mantel Cox comparison.

## Results

Of the 126 consecutive patients who underwent pancreatic resections 104 (62 male) with a median (IQR) age of 64 (59-72) years had cancer and 22 (15 male) with a median (IQR) age of 55 (41-61) years had chronic pancreatitis. Specimens of chronic pancreatitis tissue were obtained from areas adjacent to cancer in 78 patients. Normal pancreatic tissue was obtained from the resection specimens, adjacent to areas of cancer or chronic pancreatitis in a total of 30 patients. Sixty-one metastatic lymph nodes from 61 patients were also studied [16]. Of the 104 patients with cancer, 31 (15 male) had ampullary adenocarcinoma, 24 (15 male) had cholangiocarcinoma, 44 (29 male) had pancreatic ductal adenocarcinoma and 5 (3 male) had duodenal adenocarcinoma. The ratios of well:moderately:poorly differentiated tumours were 7:16:8, 1:18:5, 5:28:11 and 0:5:0 for ampullary adenocarcinoma, cholangiocarcinoma, pancreatic ductal adenocarcinoma and duodenal adenocarcinoma respectively. Vascular invasion was present in 13 (42%), 8 (33%), 22 (50%) and 1 (20%) patients with ampullary adenocarcinoma, cholangiocarcinoma, pancreatic ductal adenocarcinoma and duodenal adenocarcinoma respectively. Corresponding figures for perineural invasion were 12 (39%), 14 (58%), 30 (68%) and 2 (40%) and those for lymphatic invasion were 12 (39%), 14 (58%), 26 (59%) and 4 (80%). Tumours were ≥ 2 cm in diameter in 12 (39%), 7 (29%), 15 (34%) and 2 (40%) patients with ampullary adenocarcinoma, cholangiocarcinoma, pancreatic ductal adenocarcinoma and duodenal adenocarcinoma respectively. AICC disease stage distribution (Ia:Ib:II) was 12 (39%):11 (35%):8 (26%) for those with ampullary adenocarcinoma, 7 (29%):7 (29%):10 (42%) for cholangiocarcinoma, 15 (34%):12 (27%):17 (39%) for pancreatic ductal adenocarcinoma and 2 (40%):2 (40%):1 (20%) for duodenal adenocarcinoma.

### MMC by grade of the tumour

The immunohistochemical scores for MMCs I, III, IV and V, categorized as high or low expressors by tumour grade for various periampullary tumour types, are summarized in Table 1. Across tumour grades the ratios of high to low expressors of MMCs I, III and IV were comparable. However, for MMC V the ratios were reversed for poorly differentiated tumours compared to well or moderately differentiated tumours, i.e. poorly differentiated tumours tended to show low expression of MMC V, compared to well and moderately differentiated tumours (Table 1). Moreover, high MMC V expression showed a statistically significant inverse correlation with the number of lymph node metastases (Pearson's correlation coefficient of -0.314, 2 tailed significance of p < 0.001). Volume corrected mitotic index (VCMI) showed an inverse correlation with enhanced MMC I expression (Pearson's correlation coefficient of -0.231, 2 tailed significance of p < 0.001).

**Table 1:** Distribution by immunoscore of mitochondrial membrane complexes (MMC) I, III, IV and V among all periampullary cancers.

MMC	Immunoscore	Statistic	Degree of differentiation		
	(Range 0-12)		*Well*	*Moderate*	*Poor*
**I**	***High expressors***	No. of patients	5	25	5
	(Score >8)	Mean (SD) immunoscore	12 (0)	12 (0)	12 (0)
	***Low expressors***	No. of patients	8	42	19
	(Score ≤ 8)	Mean (SD) immunoscore	7 (2)	7 (2)	8 (1)
**III**	***High expressors***	No. of patients	6	40	14
	(Score >8)	Mean (SD) immunoscore	12 (0)	12 (0)	12 (0)
	***Low expressors***	No. of patients	7	27	10
	(Score ≤ 8)	Mean (SD) immunoscore	7 (2)	7 (2)	8 (0)
**IV**	***High expressors***	Count (no. of patients)	6	33	14
	(Score >8)	Mean (SD) immunoscore	12 (0)	12 (0)	12 (0)
	***Low expressors***	Count (no. of patients)	7	34	10
	(Score ≤ 8)	Mean (SD) immunoscore	7 (2)	7 (2)	7 (1)
**V**	***High expressors***	Count (no. of patients)	8	35	7
	(Score >8)	Mean (SD) immunoscore	11 (2)	10 (2)	9 (2)
	***Low expressors***	Count (no. of patients)	5	32	17
	(Score ≤ 8)	Mean (SD) immunoscore	2 (2)	2 (2)	2 (1)

### MMC expression by tissue type in the same tumour and by diagnosis

**Figure 1:**
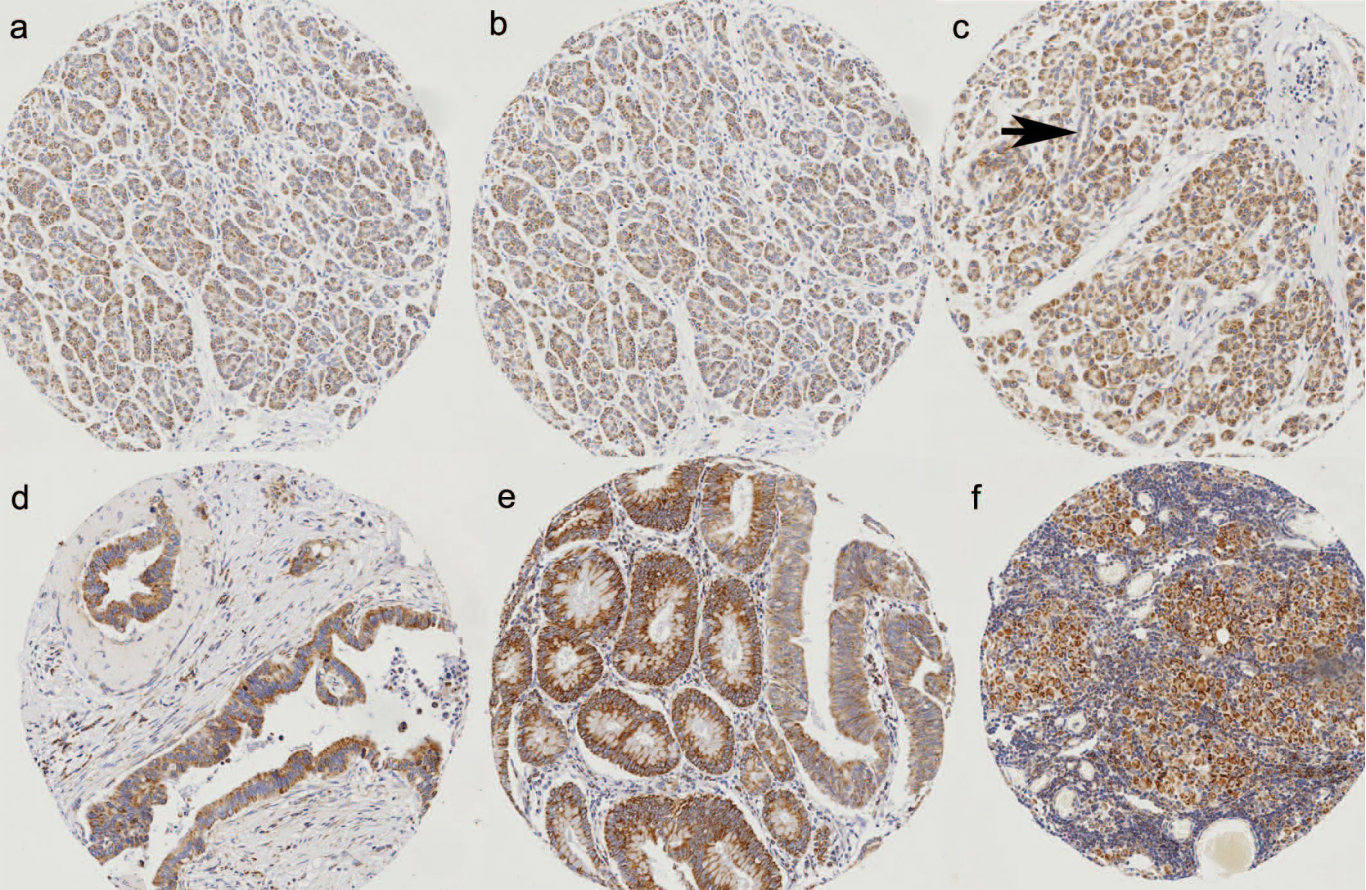
**Expression of MMC I**. (a and b) Normal pancreatic acini showing weak expression; (c) Chronic pancreatitis tissue showing weak acinar expression but no expression in the pancreatic duct (arrow); (d and e) Pancreatic cancer showing strong expression; (f) Metastatic cancer in lymph node showing strong expression (Magnification for all images ×40)

**Figure 2:**
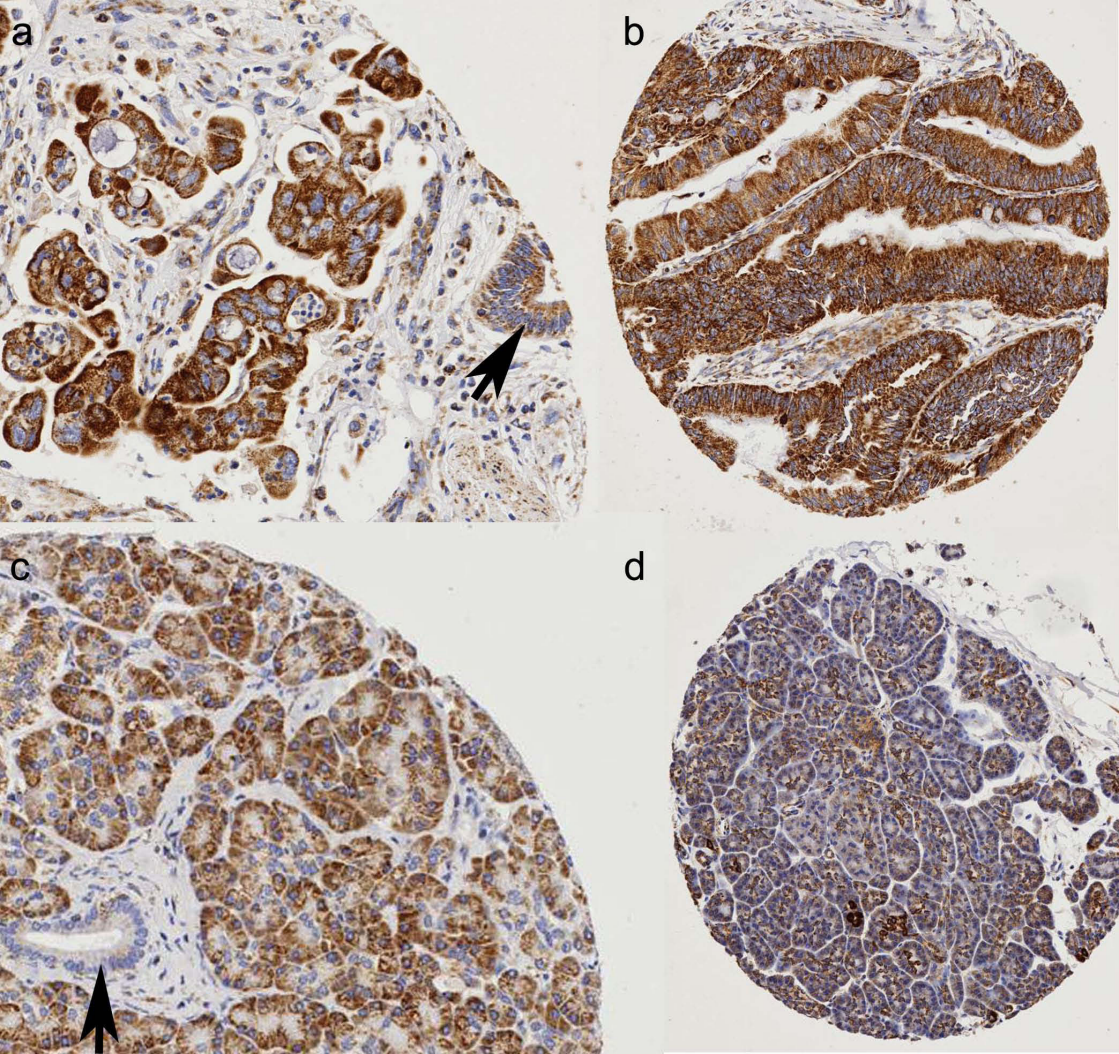
**Expression of MMC III**. (a) Strong expression in pancreatic cancer (×60); with weak expression in adjacent benign pancreatic duct epithelium (arrow); (b) Strong expression in pancreatic cancer (×40); (c) Chronic pancreatitis (×60) showing weak expression in the acini but no expression in the ductal epithelium (arrow); (d) Normal pancreas (×40) showing very weak expression.

**Figure 3:**
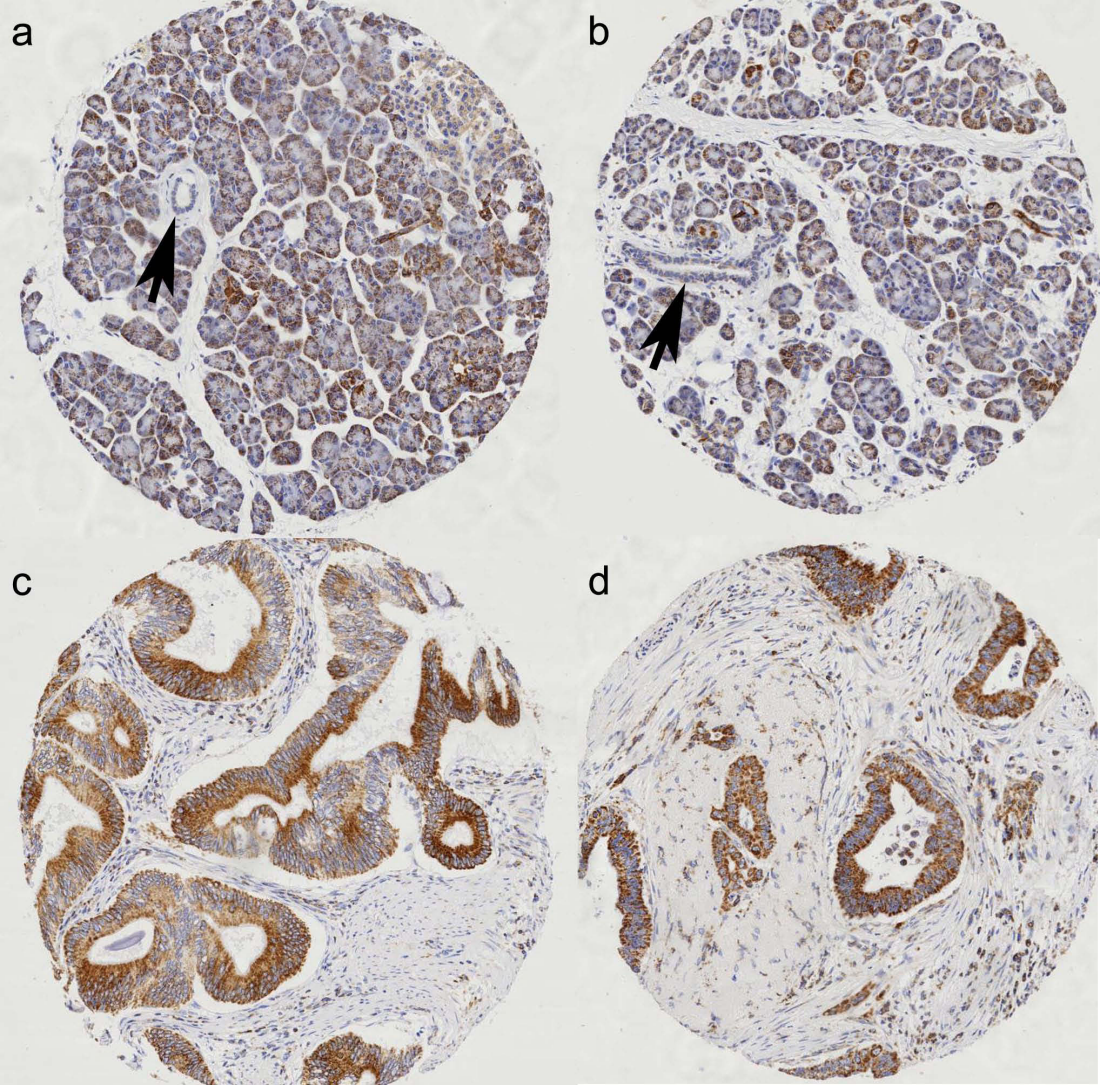
**Expression of MMC IV**. (a and b) Weak expression in benign pancreatic acini (×40); with no expression in the ductal epithelium (arrows); (c and d) Strong expression in pancreatic cancer (×40).

**Figure 4:**
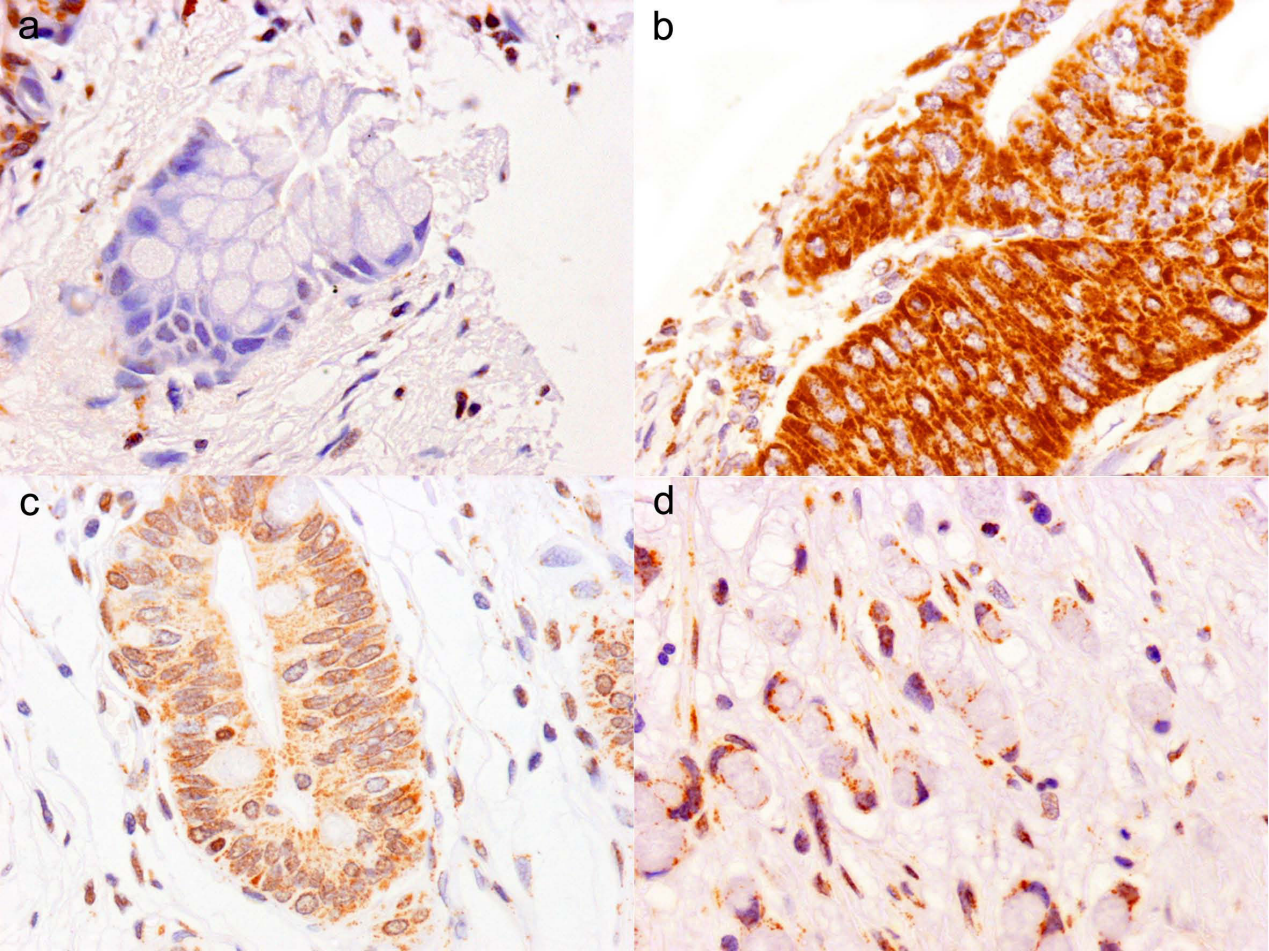
**Expression of MMC V.  **(a) Lack of expression in a benign pancreatic ductal epithelium (×80); (b-d) strong expression in pancreatic cancer (×80); (b and c) well differentiated adenocarcinoma (×80); (d) poorly differentiated adenocarcinoma (×80).

**Figure 5:**
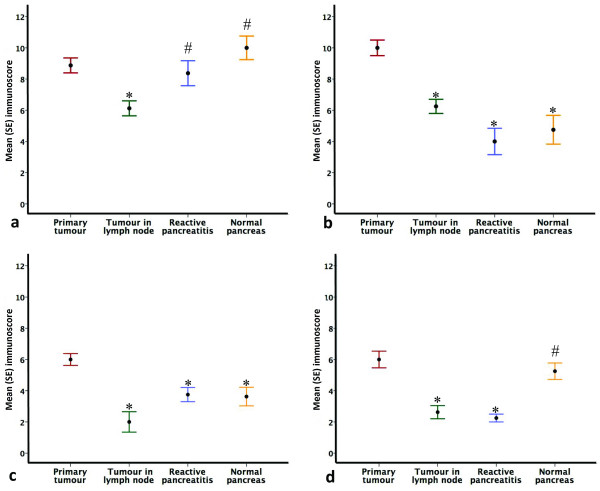
**Expression of MMCs observed in various tissues within resection specimens**. (a) Mean (SE) immunoscore across various tissue types in the same tumour specimen for MMC I; (b) Mean (SE) immunoscore across various tissue types in the same tumour specimen for MMC III; (c) Mean (SE) immunoscore across various tissue types in the same tumour specimen for MMC IV; (d) Mean (SE) immunoscore across various tissue types in the same tumour specimen for MMC V. All comparisons were made with the primary tumour (*p < 0.001, # not significant).

**Figure 6:**
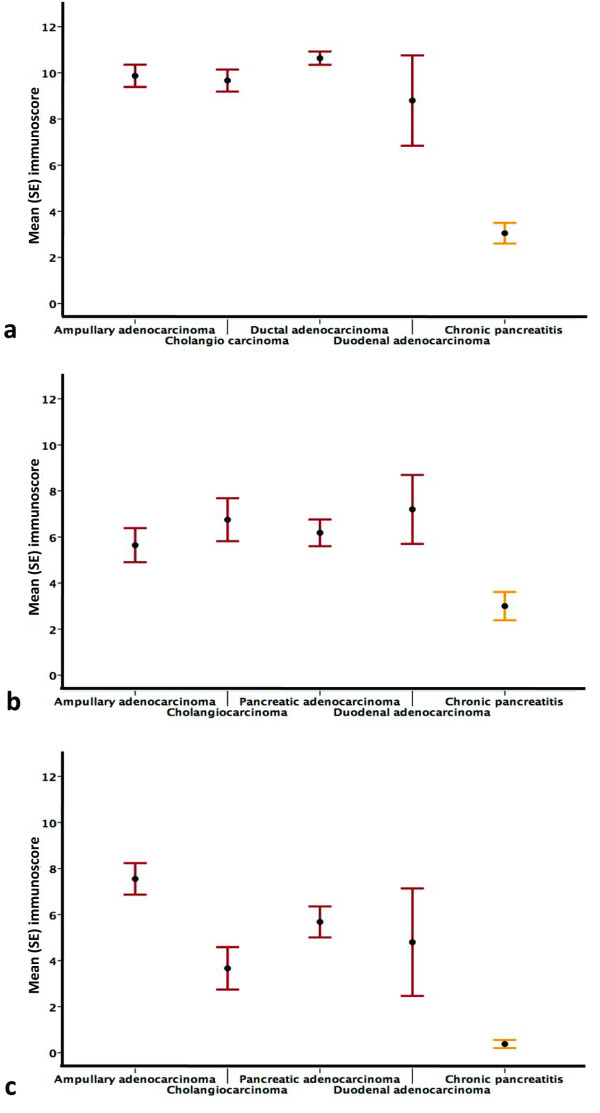
**Expression of MMCs across tumour types**. (a) Mean (SE) immunoscore across different diagnoses for MMC III, cancers, chronic pancreatitis; (b) Mean (SE) immunoscore across different diagnoses for MMC IV; cancers (5.4-7.0, 95% CI), chronic pancreatitis; (c) Mean (SE) immunoscore across different diagnoses for MMC V; cancers, chronic pancreatitis. Comparisons of each tumour type made with chronic pancreatitis were significant at p < 0.001).

MMC I expression in cancer was not significantly higher than in benign tissue. A significantly greater expression of MMCs III, IV and V was noted in cancer cells compared with surrounding benign tissue (reactive pancreatitis or normal pancreas) (Figures 1, 2, 3 and 4). The pattern of expression is summarized in Figure 5. In contrast to differential expression within tissues of the same tumour, MMC expression across a range of periampullary cancer types showed an increased expression in all of them compared with chronic pancreatitis MMCs III, IV and V (Figure 6).

**Figure 7:**
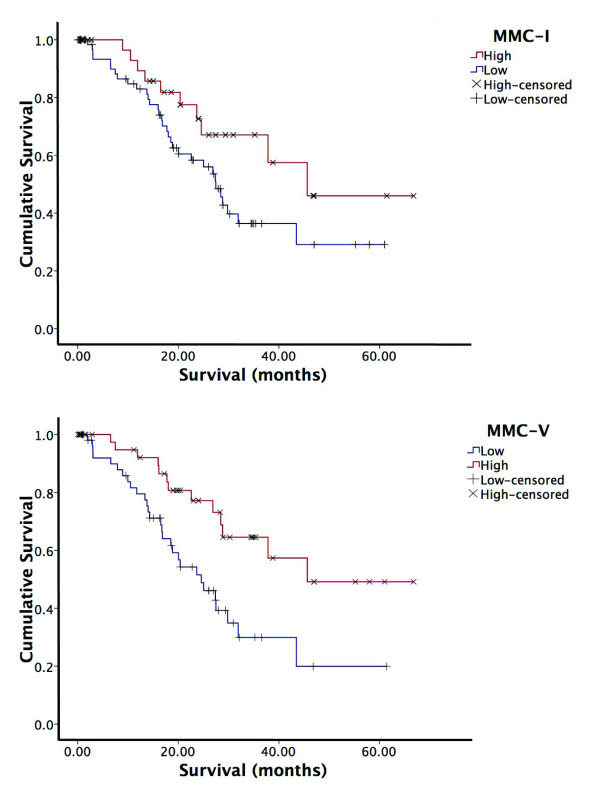
**Survival**. (a) Disease specific survival of high versus low expressors of MMC I. A trend for better survival was observed in high expressors compared with low expressors (p = 0.08); (b) Disease specific survival of high versus low expressors of MMC V. High expressors survived significantly longer than low expressors (p = 0.005).

There was a tendency to better survival in patients who showed high expression of MMC I in their cancer compared with those who had no or low expression of MMC I, although this did not reach statistical significance (Kaplan Meier, Mantel Cox p = 0.08). However, higher expression of MMC V in the cancer, was associated with better survival (Kaplan Meier, Mantel Cox p = 0.005). This may, in part, relate to lower expression of these MMCs in poorly differentiated tumours compared to well and moderately differentiated tumours (Table 1, Figure 7). In addition, high expression of MMC V showed inverse correlation with lymph node metastases (r = -0.314, p < 0.001) and high VCMI (r = -0.231, p = 0.018).

## Discussion

This study has shown that MMCs I, III, IV and V are highly expressed in periampullary cancers compared with metastatic cancer in lymph nodes or benign periampullary tissue for MMCs III, IV and V. MMC II expression was not studied because of non-availability of a suitable monoclonal antibody for immunostaining of formalin-fixed tissues. Normal ductal cells exhibit very low levels of staining, which may be to due to low levels of mitochondrial expression in these cells, and/or the limited sensitivity of the methods/antibodies used. This, to the best of our knowledge, is the first study demonstrating a differential expression of MMCs in malignant compared with benign periampullary tissue. In future studies, we will also correlate our data with the expression of the tricarboxylic acid (TCA) cycle (Krebs' cycle) enzyme citrate synthase which has been used as a marker of mitochondrial mass and may allow detection of mitochondria in ductal cells due to its high level of expression in normal cells. Cancer cells are capable of withstanding an adverse microenvironment (hypoxia, acidosis, hypoglycaemia, and shortage of growth factors) by virtue of metabolic adaptation [19]. MMCs I and III, in addition to their role as proton carriers in the electron transport chain, are known to be crucial mediators for hypoxia induced reactive oxygen production [20-22]. Such a mechanism is crucial for cellular survival in adverse hypoxic conditions. Therefore, a differentially higher expression of MMCs in primary periampullary cancer, compared with metastatic lymph nodes or benign periampullary tissue may represent an adaptive mechanism to chronic cellular hypoxia.

Periampullary cancers carry a poor prognosis due to their insensitivity to most conventional therapies [23,24]. Currently, surgical resection offers the only potential chance for a cure in these patients. The 5-year survival rate of all patients with pancreatic cancer is below 5%, and the median survival time after diagnosis is 6 months and only about 20% of patients who undergo curative resection survive longer than 5 years [25].

One mechanism of action of mitochondrial targeted anti-cancer drugs relies on their ability to disrupt the energy producing systems of cancer cell mitochondria, leading to increased reactive oxygen species and activation of the mitochondrial dependent cell death signaling pathways inside cancer cells. This emerging class of drugs called "mitocans" should act by altering properties of the mitochondria inside cancerous but not normal cells for instance by reducing the resting membrane potential by blocking the electron transport chain. It is clear from the present studies that mitocans offer great potential as effective and exciting new developments in cancer therapy, providing direct activation of cancer cell death by mitochondrial mediated apoptosis and that this complements the other pathways by which existing treatments kill cancer cells [2-5]. A proof of concept for MMC targeted therapy is the ability of clomipramine induced inhibition of MMC-III in cancer cells, as previous shown by our group [12]. Vitamin E analogues [4] have been shown to be an inhibitor of MMC II and tamoxifen, an inhibitor of MMC I [26].

Mitochondria-mediated anti-cancer therapy may also rely on manipulation of tumour suppressor proteins that are encoded by mitochondrial membrane complex genes. These genes include the succinate dehydrogenase (SDH) encoder. SDH is part of the TCA cycle that connects glucose metabolism in the cytosol to oxidative phosphorylation in the mitochondria and is the only membrane-bound enzyme of the TCA cycle and is also a functional member (complex II) of the electron transport chain. Inherited or somatic mutations in SDH have been shown to lead to the development of phaeochromocytomas and paraganglionomas [27].

However, the clinical safety and efficacy of potential anti-mitochondrial therapies in periampullary tumours lies in the selective targeting of malignant epithelium with relative sparing of benign tissue. The prudent choice of a MMC targeting agent would depend on its differential expression between malignant and benign tissue.

Enhanced expression of MMCs III and V in tumours compared with chronic pancreatitis supports their mechanistic safety when administered to diagnostically uncertain pancreatic cancers, which may turn out to be chronic pancreatitis. Moreover, high expression of some of these MMCs (I and V) may be associated with better survival, by suppressing tumour biological factors like proliferative capacity and lymph node metastasis.

## Conclusions

MMC III, IV and V targeting in periampullary cancers is mechanistically viable based on their characteristic expression profiles, indicating that such targeting strategies can be selective and therefore likely to be effective with minimal collateral damage to benign pancreatic tissue.

## Competing interests

The authors declare that they have no competing interests.

## Authors' contributions

MMA was involved with the design of the study, acquisition of data, data analysis and drafting of the manuscript. AMZ was involved with the design of the study, acquisition and interpretation of the data, and critical review of the manuscript. TEB, MI, DC-T and BJR were involved with the design of the study, interpretation of the data, and critical review of the manuscript. DNL was involved with the design of the study, interpretation of the data, critical review of the manuscript and overall supervision of the work. All authors have read and approved the final version of the manuscript.

## Pre-publication history

The pre-publication history for this paper can be accessed here:

http://www.biomedcentral.com/1471-2407/10/80/prepub
